# Supplementary material for: Considerations for Applying Entropy Methods to Temporally Correlated Stochastic Datasets
Source: Entropy (Basel). 2023 Feb 7;25(2):306. doi: 10.3390/e25020306 (PMC9955719; doi:10.3390/e25020306)
Supplement: Supplementary file 1 [file entropy-25-00306-s001.zip › entropy-2095566-supplementary.pdf]

Errors in the estimated scaling exponent, α, relative to the expected value for the ARFIMA (0, *d*, 0) models (Panels A-C) and evenly spaced averaged detrended fluctuation analysis (DFA; Panels D-F) for the three dataset lengths. Each point represents one simulated dataset. The black circles represent the mean error. The error bars represent the standard deviation. The DFA hyperparameters were: $n_{\text{min}} = 10$; $n_{\text{max}} = N/4$; $k = 26$, 37, and 47 for the three dataset lengths, respectively. Linear detrending was used; higher-order detrending did not change the results. DFA estimates were characterized by positive biases up to .1 for stationary anti-persistent processes ($\alpha < 0.5$), as well as negative biases for nonstationary persistent processes ($\alpha > 1.7$). ARFIMA modeling produced comparatively smaller biases. Moreover, ARFIMA modeling also produced more precise estimates as measured by the standard deviation across datasets. In summary, ARFIMA modeling yielded a more accurate and precise estimation of temporal correlations.

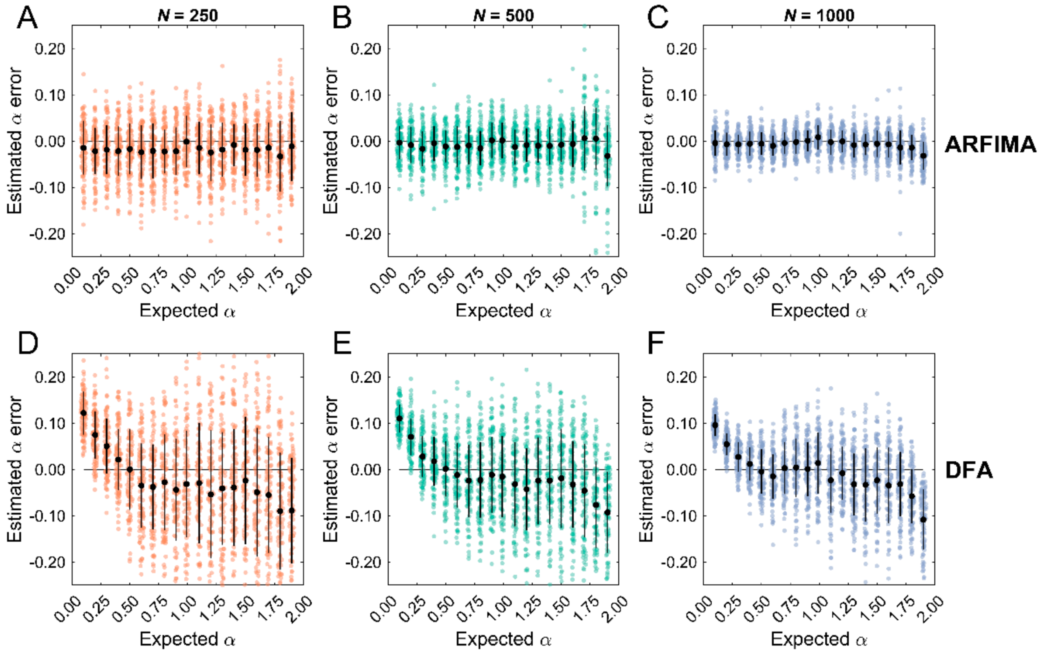

Figure S1. ARFIMA modeling provides more accurate and precise estimates than DFA.

Comparison of data classification with ARFIMA (0, d, 0) models and evenly spaced averaged detrended fluctuation analysis (DFA) for the three dataset lengths. The DFA hyperparameters were: $n_{min} = 10$, $n_{max} = N/4$, $k = 26$, 37, and 47 for the three dataset lengths, respectively. Linear detrending was used; different detrending orders did not change the results. For the short (Panel A) and medium (Panel B) dataset lengths, DFA failed to accurately classify nonstationary anti-persistent datasets $1 \leq \alpha \leq 1.2$ compared to ARFIMA modeling. This is due to the underestimation bias of DFA for nonstationary processes near the 1/f boundary (Figure S1), which has been reported previously [57–59]. This bias was reduced for the long datasets (Panel C), which led to improved data classification by DFA. However, ARFIMA modeling provided a more accurate signal classification for all three dataset lengths examined.

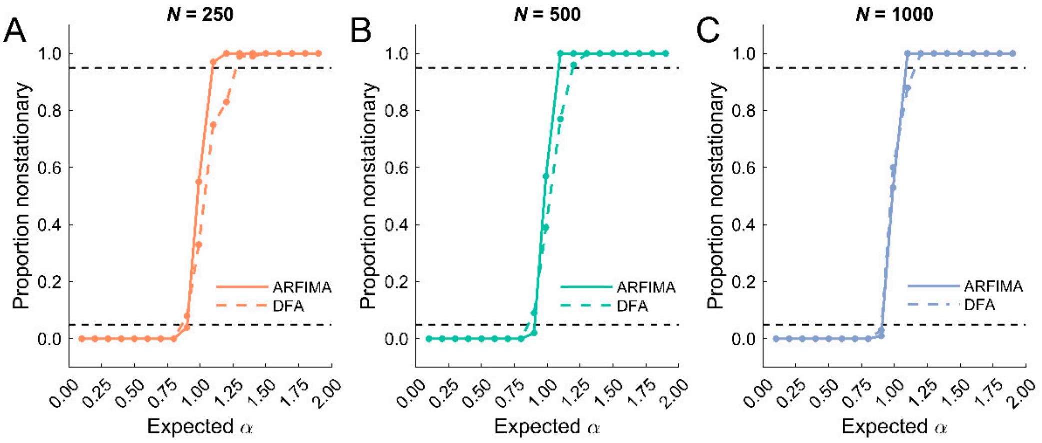

Figure S2. ARFIMA modeling provides better data classification than DFA.

To estimate the template length, m, the standard error of SampEn within and between simulated datasets was quantified for each scaling exponent, α, and dataset length, N. This allowed us to assess which m value produced the most precise SampEn estimates. We computed SampEn with $m = 1$, 2, and 3 and $r = .2$. Panels A-C display the within-simulation standard error of SampEn. Each data point represents the mean standard error across 100 simulated datasets. Larger m values produced less reliable SampEn estimates independent of α and N. The within-simulation standard error was consistently lower for the nonstationary datasets ($\alpha > 1$). Panels D-F display the between-simulation standard error of SampEn. Each data point represents the standard error of SampEn across 100 simulated datasets. Larger m values produced less reliable SampEn estimates independent of α and N. In contrast to the above, the between-simulation standard error was generally lower for the stationary datasets ($\alpha < 1$) except for the short datasets with $m = 3$ (Panel D). In summary, m had minimal impact on the precision of SampEn for nonstationary datasets. By contrast, larger m values reduced the precision of SampEn for stationary datasets. Thus, we selected $m = 1$ for all analyses to minimize variation in SampEn estimates at fixed values of α, which represent distinct temporal correlation properties. This result was consistently reproduced for different r values.

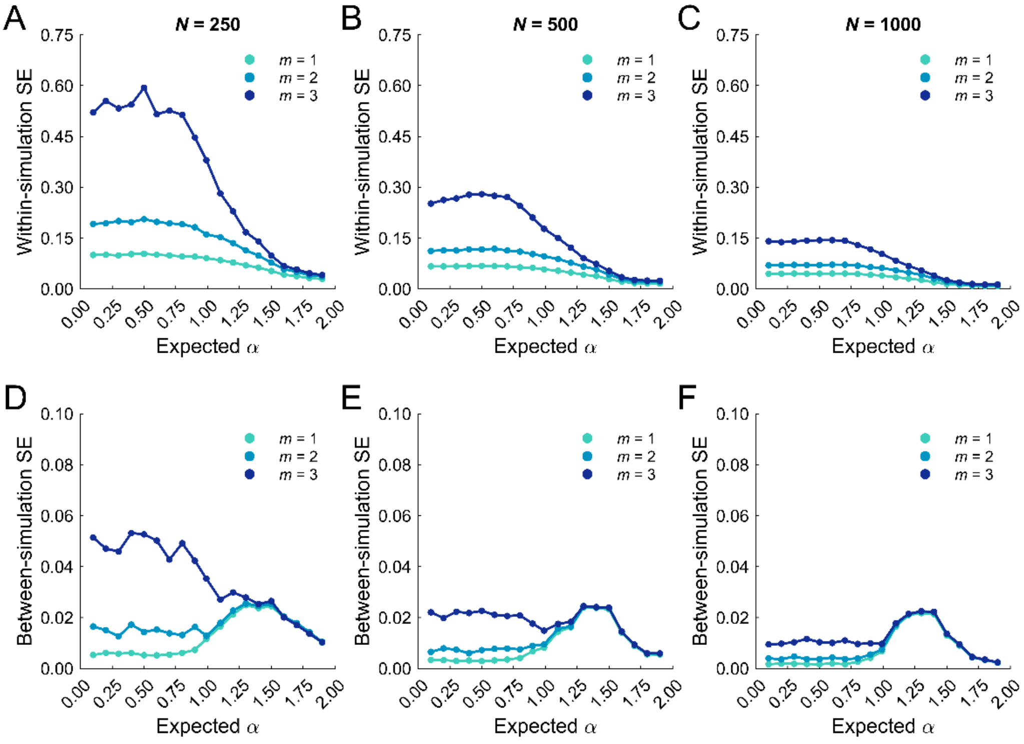

Figure S3. Estimating the template length, m.

To estimate the radius of similarity, *r*, we measured the standard error of SampEn within and between the simulated datasets for each scaling exponent, *α*, and dataset length, *N*. This allowed us to assess which *r* value produced the most precise SampEn estimates. We computed SampEn with *r* = .15, .2, .25, .3, and .35 and 3 and *m* = 1. Panels A-C display the within-simulation standard error of SampEn. Each data point represents the mean standard error across 100 simulated datasets. Larger *r* values produced more reliable SampEn estimates independent of *α* and *N*. The within-simulation standard error was consistently lower for the nonstationary datasets (*α* > 1). Panels D-F display the between-simulation standard error of SampEn. Each data point represents the standard error of SampEn across 100 simulated datasets. Larger *r* values produced more reliable SampEn estimates independent of *α* and *N*. In contrast to the above, the between-simulation standard error was consistently lower for the stationary datasets (*α* < 1). In summary, *r* had less influence on the precision of SampEn for stationary datasets. Smaller *r* values reduced the precision of SampEn for nonstationary datasets. These trends are opposite of those observed for the template length, *m* (Figure S3). There were modest improvements in the precision of SampEn for *r* greater than .25. Consequently, we selected *r* = .25 for all analyses to minimize variation in SampEn estimates at fixed *α* values without coarsening the ability of SampEn to discriminate distinct dynamical patterns. This result was consistently reproduced for different *m* values.

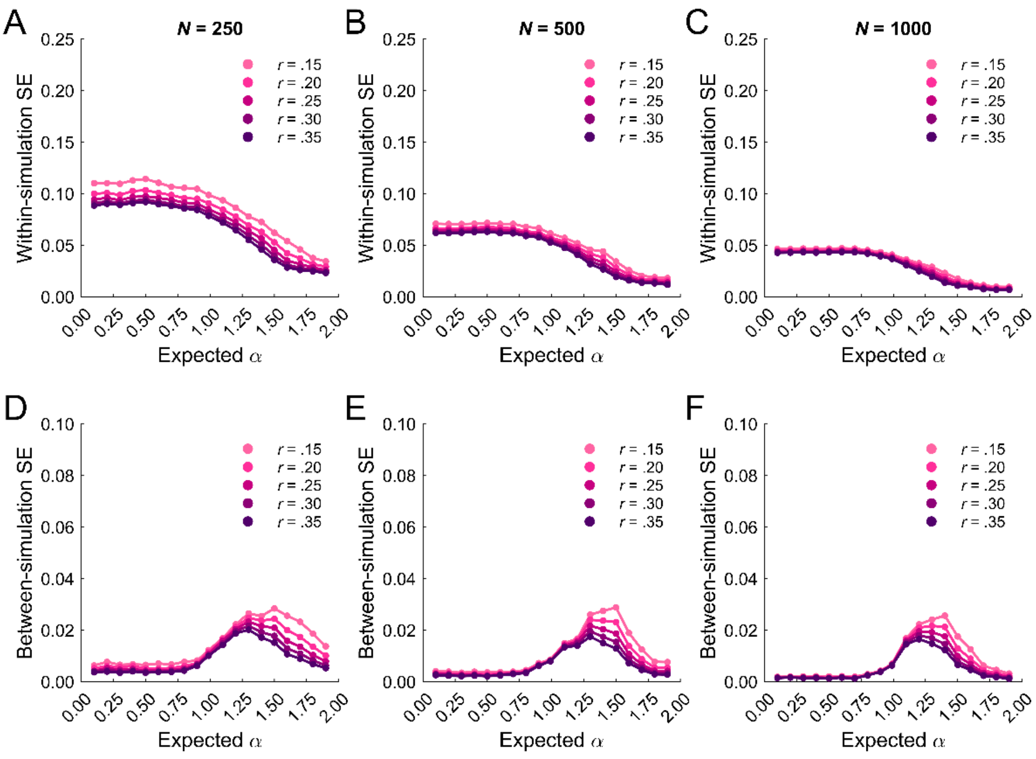

Figure S4. Estimating the radius of similarity, *r*.

To estimate the time delay, τ, the first minimum of the autocorrelation function was estimated from each simulated dataset. An alternative estimate of the first minimum of time-delayed average mutual information produced similar results. Panels A-C show the estimated time delay, τ, as a function of the scaling exponent, α, and dataset length, N. Each point represents one of the 100 simulated datasets. The black circles and error bars represent the mean and standard deviation, respectively. On average, τ estimates were close to 1 for anti-persistent processes ($\alpha < .5$) and less than 2 for uncorrelated processes ($\alpha = .5$). By contrast, τ estimates increased monotonically for persistent processes ($\alpha > .5$); the greater α, the larger the τ estimates. τ estimates became increasingly larger for the nonstationary datasets ($\alpha > 1$). The maximum allowable τ value was 50 because SampEn did not converge for higher τ values due to finite dataset lengths, which is why estimates are asymptotes for the nonstationary datasets. To examine the impact of τ on SampEn, τ was estimated, and each dataset was submitted to SampEn(m = 1, r = .25, τ). Panels D-F show the difference between SampEn(m = 1, r = .25, τ) and SampEn(m = 1, r = .25, τ = 1) for each scaling exponent, α, and dataset length, N. The black circles and error bars represent the mean and standard deviation, respectively. The red line represents zero difference. The light red patch represents 3x the standard deviation of the SampEn estimate with τ = 1. SampEn was relatively unaffected when τ varied. This was expected for $\alpha \leq .5$ because the estimated τ values were close to 1. For the stationary persistent datasets ($\alpha > .5$), the mean differences in SampEn between the fixed and variable τ estimates were less than .011 for all dataset lengths. For the nonstationary datasets ($\alpha > 1$), the mean differences were largest for the short datasets—up to .08—and decreased as the dataset length increased—less than .03 and .02 for the medium and long datasets, respectively. In summary, these results suggest that τ has a relatively minimal effect on SampEn estimates for stationary stochastic datasets. Adjusting for τ had a larger impact on short nonstationary datasets, but the differences were still relatively small on average. This suggests that τ = 1 is appropriate when examining temporally correlated stochastic datasets.

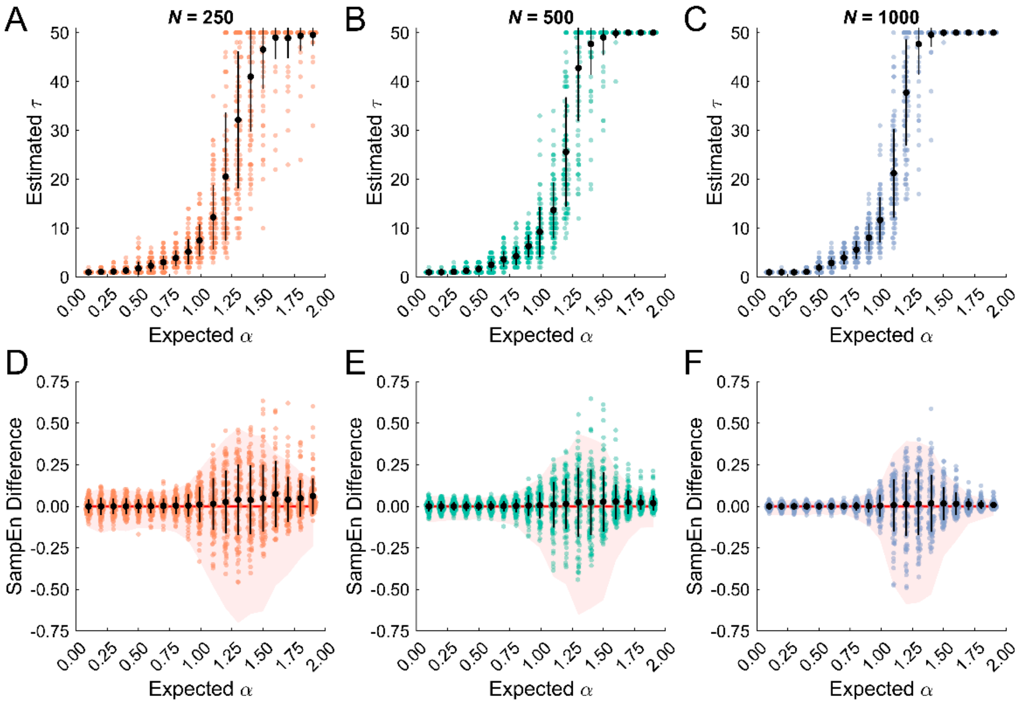

Figure S5. Estimating the time delay, τ.
